# How do parents of children with juvenile idiopathic arthritis (JIA) perceive their therapies?

**DOI:** 10.1186/1472-6882-8-25

**Published:** 2008-06-02

**Authors:** Kelly Rouster-Stevens, Savithri Nageswaran, Thomas A Arcury, Kathi J Kemper

**Affiliations:** 1 Department of Pediatrics, Wake Forest University School of Medicine, Winston-Salem, NC, USA; 2 Department of Family and Community Medicine, Wake Forest University School of Medicine, Winston-Salem, NC, USA; 3 Department of Social Science and Health Policy, Division of Public Health Sciences, Wake Forest University School of Medicine, Winston-Salem, NC, USA; 4 Department of and Epidemiology and Prevention, Division of Public Health Sciences Wake Forest University School of Medicine, Winston-Salem, NC, USA

## Abstract

**Background:**

Complementary and alternative medical (CAM) therapies are commonly used by pediatric patients with chronic medical conditions. Little is known about parents' perceptions of these therapies. This study describes the views of parents of patients with juvenile idiopathic arthritis (JIA) regarding conventional and CAM therapies.

**Methods:**

Parents of children with JIA seen at a pediatric rheumatology clinic were surveyed between June 1 and July 31, 2007. Questionnaires asked about patients' use of over 75 therapies in the past 30 days, their perceived helpfulness (0 = not helpful; 3 = very helpful), perceived side effects (0 = none; 3 = severe), and whether each therapy would be recommended to other patients with JIA (Yes, No, Not sure).

**Results:**

Questionnaires were returned by 52/76 (68%) parents; patients' average age was 10.9 years and 87% were Caucasian. Medications were used by 45 (88%) patients; heat (67%) and extra rest (54%) were also commonly used. CAM therapies were used by 48 (92%), e.g., massage (54%), vitamins and other supplements (54%), avoiding foods that worsened pain (35%) and stress management techniques (33%). Among the therapies rated by 3 or more parents, those that scored 2.5 or higher on helpfulness were: biologic medications, methotrexate, naproxen, wheelchairs, orthotics, heat, vitamins C and D, music, support groups and prayer. CAM therapies had 0 median side effects and parents would recommend many of them to other families.

**Conclusion:**

JIA patients use diverse therapies. Parents report that many CAM therapies are helpful and would recommend them to other parents. These data can be used in counseling patients and guiding future research.

## Background

Juvenile Idiopathic Arthritis (JIA), is the most common arthropathy of childhood with an estimated prevalence of 7 to 400/100,000 children [1]. Pharmacologic therapy traditionally involves the use of non-steroidal anti-inflammatory drugs (NSAIDs); modern therapy also includes disease-modifying agents, including biologic agents, methotrexate, sulfasalazine and corticosteroids. Physical and occupational therapy are integral to maintain mobility and adapt to daily living. Extra rest as well as heat and exercise are also commonly recommended to help reduce symptoms.

Complementary and alternative medical (CAM) therapy has been variously defined as including dietary supplements, dietary modifications, stress management, prayer, massage, acupuncture, homeopathy, and home or folk remedies such as dietary changes, liniments and copper bracelets [2]. As reported in previous studies, patients with chronic pain, such as those with JIA use CAM therapies [3,4]. For example, 64% of pediatric rheumatology patients in Toronto reported using CAM, most often vitamins, minerals, and relaxation techniques [5]. Most (70%) children attending arthritis youth camps in Australia, New Zealand and Canada reported using CAM therapies such as copper bracelets, dietary changes, and patent medicines [6]. In a survey of 76 patients with diverse diagnoses seen at the Wake Forest University rheumatology clinic between 2001 and 2003, the most commonly reported CAM therapies were faith healing (14%) and massage (11%) (personal communication with Dr. Sara Sinal, 1/9/07). Differing rates among these studies may have been reported due to geographic differences, differences in survey questions, differences in survey populations, and changes in practice over time. Overall, little is known about how patients' families perceive the effectiveness and safety of CAM, home and folk remedies or, for that matter, how they perceive mainstream therapies for JIA. Recent advances in understanding the pathophysiology of arthritis have broadened the medication repertoire for treating JIA. Physicians anticipate that their patients follow treatment recommendations, but parent's perceptions of these treatments will influence what the child ultimately receives. Furthermore, families often talk with one another in waiting rooms, but little is known about what they recommend to each other. Given the variety of treatments for JIA, both mainstream and CAM, it is important to assess parental perception of therapy; to our knowledge, this is the first study to address this in patients with JIA.

This study had three aims: 1) to describe the use of specific CAM therapies (including home remedies and folk remedies), in the context of their use of conventional therapies; 2) to assess parents' perceptions of the helpfulness and side effects of all therapies the patient had used recently and 3) to describe what therapies parents would recommend to other families of JIA patients. We were interested in these questions to better counsel patients and to understand which CAM therapies might be most acceptable for future study in this population. The survey was not conducted as hypothesis-testing study.

## Methods

We surveyed parents of JIA patients between June 1 and July 31, 2007. Parents were eligible if they had a child diagnosed with JIA using the International League of Associations for Rheumatology classification [7] who had been seen within the previous six months by the only pediatric rheumatologist (KRS) at Brenner Children's Hospital. This clinic cares for children from birth through 21 years of age. The patient's medical records were reviewed by a research assistant supervised by the rheumatologist to determine eligibility, patients' ages and diagnostic category. Parents were recruited through a letter sent by the rheumatologist and the director of the Program for Holistic and Integrative Medicine (KJK). The letter included the survey questionnaire, a stamped self-addressed return envelope, $2 cash, and an offer of $10 gift certificate for completion and return of the survey questionnaire. The letter asked parents to complete the surveys with the patients' assistance to enhance accuracy.

### Questionnaire

The questionnaire included demographic questions, a single question on the patient's overall health status (excellent/good/fair/poor), and information about the duration and type of the child's illness. Demographic characteristics included the patient's age, gender, race/ethnicity, maternal education and parents marital status and type of JIA. We also asked about patients' other illnesses. Questionnaires were sent to the patients' parents and were generally completed by patients' mothers (98%) with input from the patients themselves.

We assessed patients' use of different therapies with an extensive list of conventional therapies such as prescription and non-prescription medications, assistive devices, physical therapy interventions and home care (extra rest, therapeutic exercise); and complementary therapies (e.g., specific supplements, massage, magnets, stress management tools, spiritual healing, etc.); and professional care provided by chiropractors, acupuncturists, massage therapists and naturopathic clinicians. These therapies were compiled from three primary sources: previous research on CAM use in rheumatology and pediatrics; local experience in pediatrics; and statewide experience among older adults with arthritis [8]. The list was pilot tested in parents of two adolescents with JIA who were not part of this study; no revisions were suggested. For each therapy, questions addressed a) whether the therapy had been used in the past 30 days (yes/no); for those who had used it, how helpful it was (0 = not helpful; 1 = somewhat; 2 = moderately; 3 = very helpful); side effects (0 = none; 1= mild; 2= moderate; 3 = severe); and whether the parent would recommend it to other patients with JIA (Yes, No or Not Sure). The entire list of therapies is available from the authors upon request.

### Data management

All questionnaires were completed anonymously. Data were entered into a secure Microsoft Access Database which was backed up nightly. Data entry was checked by a separate assistant and further checked for reasonable ranges; a 25% sample was triple checked against original paper copies to ensure accuracy.

### Analysis

Data analysis focused on descriptive statistics. We calculated the proportion of children using each therapy. Next, because some data were not normally distributed, we calculated the median and mean rating of helpfulness and side effects for each therapy. We then calculated the proportion of parents who would recommend the therapy to other patients. Only those who used the therapy were included for the calculation of helpfulness, side effects and recommendation. For each therapy class, the proportion of children who used at least one therapy within the class, the highest mean and median rating of helpfulness and side effects for the class and the proportion of those would recommend at least one therapy were calculated. Similarly, usage, maximum helpfulness and side effects and recommendation for 'any CAM therapy' were calculated from individual CAM therapies. We used Wilcoxon signed-rank tests to compare each therapy class and 'any CAM therapy' to the conventional medication class (reference). A p-value of <0.05 was considered statistically significant. We used Stata Intercooled Version 8.2 for statistical analysis.

### Human Subjects

This study was approved by the Wake Forest University School of Medicine Institutional Review Board.

## Results

Of the 76 eligible patients, 52 (68%) parents returned completed questionnaires after two reminders had been sent. Of the 52 parents, 50 were mothers and two were fathers; most (75%) respondents were married and 62% had at least some college or a college degree. The 24 patients whose parents were non-respondents were similar to the children of respondents in terms of age 11.8 ± 3.2 years versus 10.7 ± 4.8 years; race (87% of both were Caucasian) and gender (27% versus 19% male for respondents).

The age range of the children was 2.7 years to 20.1 years with a mean of 10.9 years. Patients had been diagnosed with JIA for an average of 4.9 years and most had oligoarthritis (40%) or rheumatoid factor negative polyarthritis (29%; see Table 1). Co-morbid conditions were reported for 67% of patients, most commonly allergies (37%) and headaches (27%). Nearly 75% of parents rated their child's health as excellent or very good; this is representative of the population seen in the pediatric rheumatology clinic.

**Table 1:** Sample characteristics

**Characteristic**	**N= 52 (100%)**
**Child's Age in years – median (mean ± sd)**	10.8 (10.9 ± 4.7)
**Child Gender: Male – N (%)**	10 (19)
**Child's race/ethnicity – N (%)**	
Caucasian, not Hispanic	45 (87)
African American, not Hispanic	3 (6)
Hispanic	4 (8)
**JIA Duration in years – median (mean ± sd)**	3.3 (4.9 ± 4.0)
**JIA Subtype N (%)**	
Oligoarthritis	21 (40)
Rheumatoid-factor negative polyarthritis	15 (29)
Systemic arthritis	6 (12)
Enthesitis-related arthritis	6 (12)
Other	4 (8)
**Co-morbidity: median (mean ± sd) number of problems**	1 (1.4 ± 1.6)
None N (%)	17 (33)
Allergies N (%)	19 (37)
Headaches N (%)	14 (27)
Abdominal Pain N (%)	7 (14)
Constipation or Diarrhea N (%)	6 (12)
Acne N (%)	6 (12)
Other, includes depression, scoliosis, eczema, asthma, GERD N (%)	10 (20)
**Overall health, parental rating – N (%)**	
Excellent	13 (25)
Very Good	25 (48)
Fair	14 (27)
Poor	0

### Conventional Therapies used

The most common therapies used in the 30 days prior to the survey are listed in Table 2. Medications were used by 45 (88%) of patients, most commonly methotrexate (42%), acetaminophen (38%), and/or naproxen (33%); none used hydroxychloroquine and only two used sulfasalazine (data not shown). Eighty-eight percent used at least one medication. Most (69%) children engaged in therapeutic exercise (most commonly stretching and swimming); 67% used heat or cold for painful joints; 54% reported getting extra rest; and 20% of patients required wheelchair, orthotics, braces or splints. No patients reported using a cane or ultrasound therapy.

**Table 2:** Perceptions and recommendations about therapies used by three or more patients in past 30 days (N = 52)

**Therapy**	**Used in past 30 days n (%)**	**Helped Median (Mean)**		**Side effects Median (Mean)**		**Recommended **N (% of those who made recommendations)
***Medications***		0 = not helpful; 3 = very helpful	P value*	0 = none; 3 = severe	P value†	

***Medications (Any)***	**45 (88)**	**3 (2.7)**	*Reference*	**1 (1)**	*Reference*	**40 (87)**
Methotrexate	22 (42)	3 (2.5)		0 (0.7)		17 (77)
Acetaminophen	20 (38)	2 (1.6)		0 (0.1)		15 (75)
Naproxen	17 (33)	3 (2.4)		0 (0.6)		12 (80)
Ibuprofen (any brand)	14 (27)	2 (1.6)		0 (0.2)		10 (77)
Biologics: Etanercept, Adalimumab, or Infliximab	10 (19)	3 (3.0)		0 (0.2)		8 (80)
Celebrex^®^, Mobic^®^, Relafen^®^, or Talmentin^®^	7 (13)	3.0 (2.1)		0 (0.6)		4 (57)
Corticosteroids (prednisone or prednisolone)	8 (15)	2.5 (2.1)		1 (1.4)		2 (29)
Diclofenac (Voltaren^®^)	6 (12)	2.5 (2.2)		0 (0.6)		4 (80)
***Physical Therapy***	**12 (23)**	**2 (1.9)**	*0.09*	**0 (0.3)**	*0.04*	**11 (92)**
***Heat or cold (Any)***	**35 (67)**	**2 (2.2)**	*0.01*	**0 (0.23)**	*0.0001*	**31 (94)**
Hot baths or hot tub (to ease pain)	28 (56)	2.5 (2.2)		0 (0.1)		27 (100)
Ice packs	14 (27)	1 (1.2)		0 (0.6)		9 (64)
Hot packs	13 (25)	2 (2)		0 (0.1)		11 (85)
***Assistive Devices (Any)***	**10 (20)**	**2.5 (2.2)**	*0.23*	**0 (0.7)**	*0.35*	**9 (90)**
Wheelchair	4 (8)	3 (2.8)		1 (1.3)		3 (75)
Orthotics (shoe inserts)	4 (8)	3 (2.5)		0		4 (100)
Braces	4 (8)	1.5 (1.8)		0.5 (0.5)		2 (50)
Splints	3 (6)	2 (1.7)		1 (1)		2 (67)
***Therapeutic exercise (Any)***	**36 (69)**	**1 (0.69)**	*0.008*	**0 (0.48)**	*0.06*	**32 (89)**
Stretching	29 (56)	2 (2)		0 (0.4)		24 (86)
Swimming	27 (52)	3 (2.2)		0 (0.3)		23 (92)
Other: aerobics, exercise ball, cheerleading, biking	6 (12)	3 (2.5)		0 (0.4)		5 (83)
***Extra sleep or rest***	**28 (54)**	**2 (2)**	*0.0006*	**0 (0.2)**	*0.002*	**25 (89)**
**ANY CAM, including HOME OR FOLK REMEDY LISTED BELOW**	**48 (92)**	**3 (2.5)**	*0.29*	**0 (0.29)**	*0.002*	**41 (91)**
**Massage **by self or family member	**28 (54)**	**2 (1.9)**	*0.03*	**0 (0.3)**	*0.003*	**22 (81)**
***Salves/ointments ***: Vicks Vapo-Rub	**3 (6)**	*2*	**0.16**	**0 (0.7)**	*0.32*	**3 (100)**
***Vitamins and other Dietary supplements (ANY)***	**28 (54)**	**2 (1.9)**	*0.03*	**0 (0.12)**	*0.002*	**22 (88)**
Multivitamins	21 (40)	2 (1.9)		0 (0.2)		15 (83)
Vitamin C	10 (19)	2.5 (2.1)		0		9 (100)
Vitamin D	7 (13)	3 (2.3)		0 (0.3)		7 (100)
Fish oil or omega 3 fatty acids	3 (6)	2 (2)		0		2 (100)
Glucosamine	2 (4)	2 (2)		0		2 (100)
***Avoiding foods *that worsen pain**	**18 (35)**	**2 (1.6)**	*0.03*	**0 (0.07)**	*0.03*	**13 (18)**
Dairy (milk)	18 (35)	1.5 (1.4)		0 (0.1)		12 (67)
Meat	14 (27)	1 (1.3)		0		5 (36)
Oranges	9 (17)	1 (1.3)		0		6 (67)
Seafood/fish	9 (17)	1 (1.2)		0		5 (56)
Wheat/gluten	5 (10)	0.5 (0.8)		0		1 (20)
Tomatoes, peppers, eggplant	4 (8)	0.5 (0.8)		0		2 (50)
Soy	1 (2)	1 (1)		0		1 (100)
***Stress management***	**17 (33)**	**3 (2.5)**	*0.13*	**0**	*-*	**14 (82)**
Music	12 (23)	2.5 (2.3)		0		9 (75)
Support Groups	6 (12)	3 (2.8)		0		5 (83)
Relaxation tape or CD	6 (12)	2.5 (2.5)		0		4 (67)
**Spiritual *Healing (Prayer)***	14 (27)	3 (2.6)	*0.69*	0 (0.1)	*0.004*	13 (93)

* p values for helpfulness for each therapy are calculated with medications (any) as the reference.

† p values for side effects for each therapy are calculated with medications (any) as the reference

### Complementary therapies used

Complementary therapies were used by 92% of patients (see Table 2). For example, 54% of patients received massage for painful joints. Unlike the studies in adults [9-11], no parent reported using topical capsaicin, DMSO, olive oil, peanut oil, turpentine, vinegar, WD40^® ^or TigerBalm^® ^for their child; however, three parents reported using Vicks VapoRub^® ^on patients' joints.

Dietary supplements and dietary changes were among the most commonly used complementary remedies. For example, 40% received multivitamins and 19% received vitamin C. Fewer received vitamin D (13%), omega three fatty acids/fish oil supplements (6%) or glucosamine (4%). Unlike surveys of adult arthritis patients, no parent reported giving their child bromelain, flax seed oil, gamma linolenic acid, evening primrose oil, quercetin, S-adenosylmethionine, turmeric or curcumin. Many (35%) parents reported that they avoided giving their child specific foods that they thought might aggravate their child's pain. For example, 35% avoided dairy and 27% avoided meat; between 10% and 20% avoided oranges, fish/shellfish, or wheat/gluten. Fewer than 10% avoided tomatoes, peppers, eggplant or soy.

Stress management techniques and spiritual healing were also commonly used, but professionally provided CAM therapies were not. Between 12% and 25% of parents used music, support groups or relaxation tapes or CDs to help their child's symptoms. Slightly more than one quarter reported using spiritual healing. Fewer than 3% used guided imagery, meditation, progressive relaxation, aromatherapy, yoga, tai chi, homeopathy or magnets; only one parent each reported trying a professional counselor or Healing Touch. No parents reported using copper bracelets, biofeedback or hypnosis to help with the child's pain or other symptoms. No parent reported taking their child to an acupuncturist, chiropractor, herbalist, professional massage therapist, naturopathic clinician, or osteopathic physician in the 30 days prior to the survey.

### Perceived helpfulness and side effects

The therapies that were used most often were generally rated as the most helpful. Among medications, methotrexate, naproxen, biological agents, and prescription anti-inflammatory medications were rated as most helpful (median score of 3). Other conventional recommendations that had median scores ≥ 2.5 included corticosteroids, diclofenac, wheel chairs, orthotics, hot baths, and swimming. Although acetaminophen and ibuprofen were commonly used, they were reportedly less effective; ice was the least effective of the conventionally recommended therapies. Conventional therapies had a median side effect score of 1; the only conventional therapies for which parents reported notable side effects were corticosteroids, wheel chairs and splints. Details on what parents perceived as side effects were not collected.

Complementary remedies that were rated as most effective (median score of 3) were support groups, spiritual healing, rubbing Vicks' VapoRub^® ^on the joints, and vitamin D; those with a median score ≥ 2 included dietary supplements (multivitamins, vitamin C, fish oil and glucosamine); relaxation tape or CD and music. All complementary therapies had a median side effect score of 0.

The mean perceived helpfulness of the conventional medication class was significantly higher than that of heat or cold (p = 0.01), therapeutic exercise (p = 0.008), extra sleep or rest (p < 0.0006), vitamins and other dairy supplements (p = 0.03), and avoiding foods (p = 0.03; see Table 2). However, when comparing perceived helpfulness of the conventional medication class to 'any CAM therapy,' there was no significant difference (p = 0.29).

The mean perceived side effects of the conventional medication class was significantly higher than that of physical therapy (p = 0.04), heat or cold (p = 0.0001), extra sleep or rest (p = 0.002), massage (p = 0.003), vitamins and other dietary supplements (p = 0.002), avoiding foods (p = 0.03), and spiritual healing (p = 0.004). Perceived side effects of conventional medication was significantly higher than 'any CAM therapy' (p = 0.002).

### Parental recommendations

Parents frequently reported that they would recommend specific therapies to other parents of children with JIA; these recommendations generally mirrored the therapies' perceived helpfulness and side effect profile (see Table 2). For example, the therapy most commonly recommended to other parents was hot baths (N = 27), followed by extra rest (N = 25), stretching (N = 24) and swimming (N = 23). Methotrexate and non-prescription analgesics were recommended by most patients who used them. Fewer parents recommended avoiding therapies. For example, three parents each recommended avoiding acetaminophen and ice packs (not effective); and three recommended avoiding corticosteroids and prescription anti-inflammatory medications (side effects). No parents recommended avoiding any assistive devices, exercise, rest, joint rubs, individual vitamins or supplements, dietary changes, stress management, homeopathy, aromatherapy or Healing Touch. At least one therapy in the conventional medication class and 'any CAM therapy' class was recommended by 87% and 91% respectively among those who used them.

## Discussion

This study builds on previous research on CAM use among JIA patients internationally and adult arthritis patients in North Carolina. It extends previous findings in two ways: first, by providing a benchmark of mainstream therapies, and second by providing parents' perspectives on the helpfulness, side effects and recommendations about these therapies. Unlike earlier studies, it focuses on parental perceptions of therapies rather than demographic risk factors for using CAM or for poor adherence to conventional recommendations [4,5,12,1]. It provides rich guidance for clinicians in discussing a variety of CAM therapies in addition to medications, physical therapy and assistive devices. It also provides essential information for researchers interested in evaluating the effectiveness of specific CAM therapies. Prior to embarking on randomized, double-blind placebo controlled (RDBPC) trials to scientifically evaluate CAM therapy effectiveness, it is beneficial to have a benchmark. This study suggests that vitamin D, vitamin C, fish oil and glucosamine were CAM therapies rated by parents as effective, and these therapies may be pursued in future RDBPC trials.

As expected among patients seen in a tertiary care, pediatric rheumatology clinic, most patients in this study used medications [14]; they also frequently used conventional therapies such as physical therapy, heat, rest, and exercise [15]. Bone health is critical to address in managing children with JIA; traditionally, calcium and vitamin D have been regarded as CAM therapy, yet inquiring about calcium and vitamin D intake in these patients is routine. As the distinction between mainstream and CAM therapies has become somewhat blurred, in this study, parents were asked about specific therapies in groups (such as medications, dietary changes, stress management) rather than as mainstream versus CAM [8]. Likewise, clinicians may find it more useful and revealing to ask patients about their use of specific therapies used rather than asking about CAM therapies per se.

### Variations in CAM use

Although this study does not specifically address changes in use of therapies over time, based on previous reports, it appears that there may be variability affected by time, geography, ethnicity, and by the way in which these therapies are defined. Such changes are reflected in the use of copper bracelets; copper bracelets were the most commonly used unconventional remedy in a survey of children attending arthritis youth camps in Australia, New Zealand and Canada in 1990, but none of the parents in our 2007 study reported using them for their children [6]. Similarly, aquatic therapy has been recently reported for patients with JIA, but was not specifically included in earlier surveys [15].

Geographic variations are exemplified by Feldman's 2004 study of CAM use in Canadian JIA patients among whom naturopathy and acupuncture were the most commonly used CAM therapies, whereas these therapies were used by none of our patients; on the other hand, spiritual healing was common in this population and uncommon in Canada. Similarly, chiropractors were used by 21% of the JIA patients in Hagen's study in Toronto, but none of the patients in this study. None of the Canadian patients reported using Vick's VapoRub^® ^on their joints; this ointment was invented in North Carolina.

In a survey of Hispanic JIA patients in Chicago, the most commonly used CAM therapies were prayer and massage therapy, whereas in our largely Caucasian population, the most common CAM therapies were massage, vitamins and other supplements, avoiding foods that aggravated symptoms and stress management techniques [16].

Multivitamins and supplemental calcium and vitamin D have become commonplace recommendations for patients with JIA, yet they are included as CAM therapies in earlier surveys [5].

### Similarities and differences with adults

The pediatric patients in the present study show several similarities in the use of therapies to North Carolina adults [9], but little similarity to adults from another region [10] or adults nationally [11]. For example, like NC adult arthritis patients, over 80% of the JIA patients used medications. A similar proportion of our JIA patients and NC adults with arthritis also used rest and heat to treat symptoms. However while only 6% of the JIA patients in this study used salves, 18% of NC adults with arthritis used lotions or creams, and 52% used ointments or liniments. The complementary therapies addressed in the different studies varied. More importantly, questionnaire items for the present study asked about therapies used in the past 30 days, while in the other studies questionnaire items asked about "ever", undefined current use [9,10], or use in the past year [11].

### Parental perceptions

Few studies have examined parents' perceptions about the helpfulness or side effects of complementary or mainstream therapies for children with chronic conditions [17-19]. In a study of parents of children seen with attention problems at a tertiary care center in Boston, Chan, et al. found that 24 parents would recommend CAM therapies such as music, dietary modifications, sensory integration and exercise while only 8 parents would recommend stimulant medications [20]. When asked what therapies they would recommend other parents avoid, 7 parents warned about prescription medications and 7 warned about "unproved" therapies such as blue-green algae and magnets. Many more of the parents in our study recommended medications, and fewer recommended that parents avoid them. Interest in CAM therapies and parental recommendations to use them were common in both the ADHD and JIA groups.

Patients' parents in this study reported that their medications were effective, and that simple measures such as hot baths, rest, swimming, stretching, massage, music, guided imagery, prayer, support groups, relaxation CDs and certain vitamins were also moderately effective and free of significant side effects.

Interestingly, there was no significant difference in perceived helpfulness of conventional medication compared to 'any CAM' class. Our observation should be interpreted cautiously-there were more individual therapies included in the 'any CAM' group, thereby increasing the likelihood of perceived helpfulness of at least one therapy within that group. Furthermore, recent advances in understanding the pathophysiology of arthritis have resulted in biologic therapies demonstrating efficacy in JIA, even in patients refractory to methotrexate [21,22]. Non-steroidal anti-inflammatory drugs (NSAIDs) have been used for several years for analgesia, but they do not modify disease progression.

Although there were more therapies for 'any CAM,' perceived side effects of the conventional medication class was significantly higher. This is not surprising, as all medications are associated with adverse effects, while several of the therapies in CAM have virtually no side effects.

Dietary changes, such as restricting certain foods, were more common than expected and offer an intriguing area for future research. Dietary restrictions are also important for clinicians to address, particularly given the increased risk of osteoporosis in the JIA population [23,24]; for patients who choose to limit dairy intake to reduce symptoms, supplemental calcium, vitamin D and weight-bearing exercise may be particularly important in reducing the long-term risks of osteoporosis. Furthermore, parental perceptions about the helpfulness of vitamin D in this study support other clinical observations that vitamin D inhibits inflammatory processes and suppresses the enhanced activity of immune cells that take part in the autoimmune reaction [25]. The possibility that vitamin D might offer acute benefits on inflammatory symptoms as well as longer-term benefits on bone health combined with families' interest in this therapy suggest that this is a fertile area for future research.

### Limitations

This study had several limitations and strengths. Although it was conducted in one university-based clinic, it had a high response rate; patients in this clinic are similar demographically and in terms of disease to many others in the US. Generalizability is limited because of geographic variations in the licensing and practice of CAM clinicians; for example, as in most of the US, naturopaths are not licensed in NC, but they are licensed in some states, and children in these states might be expected to visit them [26].

Questionnaires may lead to recall bias; we attempted to minimize this and enhance accuracy by inquiring about therapies used in the past 30 days. This time frame is shorter than other studies, and may have missed therapies that had been tried and discontinued for various reasons.

We were concerned that the length of the questionnaire would prove challenging, but completion rates were high and the response to questions on recommendations were consistent with the responses to questions about helpfulness and side effects. Although we asked parents to seek input from their children when completing the questionnaire, we did not monitor questionnaire completion, so we do not know the extent to which parents did so.

This study did not collect data from the rheumatologist about treatment recommendations or determine adherence; instead it focused on parental perceptions about effectiveness and safety of therapies that had been used. Because the survey was anonymous, we cannot correlate parental reports about use of different therapies with rheumatologist recommendations.

### Future Research

The survey focused on the patients and includes some demographic data about parents, but no psychological data about parents; future studies might evaluate the impact of parental functioning on their use of CAM therapies for children with JIA. We asked parents which therapies they "would recommend" to other parents, but did not ask them how often they had actually made such recommendations; this might be an interesting question for a future study. Since the study was exploratory in nature, we used multiple testing without correction to compare each therapy class with the conventional medication class. This could have resulted in Type I error. The lack of difference seen between groups could have resulted from insufficient power. Future studies might make these comparisons using a larger sample size and a smaller number of commonly used therapies.

## Conclusion

Pediatric patients with JIA seen in a university rheumatology clinic frequently use complementary therapies in addition to their medications. Even though parents viewed some conventional medications as being more helpful than some CAM therapies overall, the use of CAM was perceived as being similarly helpful as conventional medications. Use of professionally provided CAM therapies is rare. Future studies of CAM therapies, particularly those that parents feel are helpful and low in side effects, such as vitamin D are warranted.

## Competing interests

The authors declare that they have no competing interests.

## Authors' contributions

KR–S obtained consent from all patients for the study, helped design the questionnaire, reviewed all medical records, analyze the data, draft and revise the manuscript. KJK conceived of the project, designed the overall study questionnaire, supervised data collection, entry, and analysis, drafted the manuscript and participated in revisions. TAA provided materials to help draft the study questionnaire, helped draft and edit the manuscript. SN analyzed the study data and assisted in preparation of study tables and the manuscript. All authors read and approved the final manuscript.

## Pre-publication history

The pre-publication history for this paper can be accessed here:


